# An Analysis of the Influence of DSC Parameters on the Measurement of the Thermal Properties of Phase-Change Material

**DOI:** 10.3390/ma17235689

**Published:** 2024-11-21

**Authors:** Maotiao Gao, Shiqi Zhao, Hongjun Yang, Xuehong Wu, Yingjie Xiao

**Affiliations:** 1 College of Ocean Engineering and Energy, Guangdong Ocean University, Zhanjiang 524003, China; gmt2390@163.com (M.G.); shiqizhao@163.com (S.Z.); hong0919@163.com (H.Y.); 2 School of Energy and Power Engineering, Zhengzhou University of Light Industry, Zhengzhou 450002, China; xuehongwu@163.com; 3 School of Mechanical Engineering, Guangdong Ocean University, Zhanjiang 524003, China

**Keywords:** DSC, paraffin, experiment conditions, thermal properties

## Abstract

A differential scanning calorimeter (DSC) is widely used for measuring the thermal properties of phase-change materials (PCMs). Optimizing test conditions based on material characteristics is essential for accurate results. This study investigates the effects of experimental parameters, including sample mass, heating rate, measurement modes, and atmosphere flow rate, on the phase-change enthalpy and phase-change temperature results. The findings indicate that variations in sample mass and heating rate lead to significant changes in phase-change temperatures, while an increase in purge gas flow rate reduces the phase-change enthalpy of the PCM. Based on the measurements, this study optimizes the DSC parameters and provides a reference for the accurate measurement of paraffin-based phase-change materials.

## 1. Introduction

In recent years, under the backdrop of energy shortage and the push for “green” energy, phase-change energy storage (PCES), as an effective means to solve the energy mismatch of supply and demand in space and time, has become a hot topic in the industry. Incorporating energy storage systems into conventional energy infrastructures can effectively enhance the rational management and application of energy through storage technology. Phase-change materials (PCMs) offer higher energy storage density compared to conventional sensible heat storage methods and can store and release energy with smaller temperature differences. As a result, PCMs are regarded as a key technology in the advancement of energy storage systems, promoting the development and stable utilization of renewable energy [1,2]. As the core of PCES technology, have gained significant attention from researchers both domestically and internationally. Their applications span a wide range of industries, including construction [3], cold chain [4], solar energy [5,6], packaging [7], electronics [8,9].

To enhance the development and utilization of PCMs, it is essential to study their thermal properties, including phase-change temperature, latent heat, and their phase-transition process, which provide a theoretical basis for the practical application of PCMs [10,11,12]. A DSC (differential scanning calorimeter) is the most commonly used tool for thermal analysis in material characterization experiments [11,13,14]. The thermal properties of the phase-change process, such as phase-change temperature and phase-transition enthalpy, are studied by observing the difference in heat flow between the sample and reference material as a function of time or temperature under the same temperature program by heat-flux DSC. The DSC is one of the essential instruments for the design and optimization of PCMs. Therefore, accurately determining the thermal properties of PCMs requires a deep understanding of the DSC, instrument, and accurately setting the measurement parameters based on the material’s characteristics is crucial [15].

Nazari Sam et al. [16] studied the characteristics of bio-based and paraffin-based PCMs and their applications in buildings. They investigated the thermal properties of these materials using DSC measurements. It was specifically noted that, during material characterization, the sample size and its representativeness, heating rate, and thermal conductivity of the materials can affect the test results and lead to objective errors in the measurements. Typically, the DSC manufacturer provides a set of general testing procedures. Al-Qatami et al. [17] believe that following the experimental procedure and placing the sample back into the furnace each time after adding it will result in different positions of the sample pan, thereby affecting the heat-flow signal and causing significant uncertainty in the measurements. Kahwaji et al. [15] discussed the inaccuracies in the reporting of thermal properties in reviewing the literature on the thermal properties of PCMs, and suggested that proper attention and an assessment of measurement accuracy are needed in experiments.

Different types of phase-change materials (PCMs) are influenced by various factors during thermal performance measurements using a heat-flow DSC. It is essential to adjust the instrument’s measurement parameters based on the material characteristics to ensure that the deviations of the measured thermal performance parameters remain within acceptable limits. Studies indicate that appropriate heating rates and sample quality are crucial for obtaining accurate results [18,19]. Kahwaji et al. [15] provided recommendations for the DSC measurements of PCMs to obtain accurate values for the following three important physical properties: phase-transition temperature, melting enthalpy, and heat capacity. These recommendations include regular and frequent equipment calibration, setting a heating rate not exceeding 10 K/min, and considering the onset temperature of the phase transition as the melting point of the material. The heat capacity value is significantly influenced by the contact conditions between the sample and the sample pan, as well as between the contact pan and the heater. Christy et al. [20] conducted multiple DSC measurements using the heat-flux method to analyze the impact of testing parameters, emphasizing that suitable measurement parameters are vital for accurately obtaining the enthalpy and onset temperature values of n-dodecane and n-hexadecane PCMs. This complements the findings of Barreneche et al. [21], who explored the effects of DSC operating modes on the thermal performance testing of PCMs, identifying applicable modes for different types of PCMs. Their results showed that dynamic and distribution modes had minimal effects on the test results for paraffin-based materials. Furthermore, Jin et al. [22] found that the melting and solidification processes of PCMs exhibited asymmetry under varying heating rates, measurement modes, and PCM types. However, due to the relatively small degree of undercooling during the solidification of paraffin-based PCMs, these two phase-change processes can be considered symmetrical in practical measurements and analyses. Zuo et al. [23] synthesized and analyzed the data of the peak temperatures and onset temperatures of energetic materials at different heating rates and proposed standardizing the thermal decomposition threshold temperatures across various heating rates. By fitting the data, they concluded that the Pow2P2 (two-parameter power function) can be used as the optimal function for predicting peak and onset temperatures.

To study and evaluate the impact of thermal lag in DSC measurements DSC measurement on thermodynamics, Svoboda [24] increased the thermal resistance by inserting polytetrafluoroethylene (PTFE) disks of varying thickness between the DSC test platform and the sample. The increase in thermal resistance resulted in a reduction in crystallization enthalpy by more than 30% and a decrease in the apparent activation energy of the crystallization process by over 20%. The increase in thermal resistance had a minimal effect on peak asymmetry. Rady [25] conducted a study on the thermal performance of encapsulated phase-change materials (EPCMs) using DSC instruments and found that higher heating rates cause the thermo-chemical imbalance. Günther et al. [26] put forward a suitable measurement method for EPCMs to control the measurement accuracy through experimental verification, which makes the experimental results more closely resemble the functional relationship between the phase-change enthalpy and phase-change temperature. Collectively, these studies underscore the importance of optimizing measurement parameters and methods for accurately assessing the thermal performance of phase-change materials.

The sample mass, heating rate, measurement mode, and the thermal resistance between the DSC test platform and the sample have a significant influence on the thermal properties and measurement results using DSC. In our previous research on the preparation and thermal properties of paraffin-based composite PCMs [27], we found that the experimental conditions and instrument parameter settings throughout the DSC testing process can affect the measurement results. However, other studies have not provided practical experimental verification or analysis regarding the influence of parameters throughout the entire experimental process on the measurement outcomes. In this paper, the thermal properties of paraffin waxes are measured by changing the sample mass, heating rate, and the atmospheric flow by heat-flux DSC. Moreover, the phase-transition enthalpy, *T_eim_*, *T_pm_* and *T_efm_* are also compared and analyzed, which provides a reference value for selection in experimental conditions and the engineering application for the thermal performance analysis of paraffin by DSC.

## 2. Experimental Section

### 2.1. Materials and Equipment

Paraffin (OP5E) was purchased from RUBITHERM (Berlin, Germany). The phase-transition process and phase-transition characteristic parameters of the materials, such as the onset temperature, peak temperature, and the enthalpy of phase change, were measured using a differential scanning calorimeter (DSC 214 type). The contact angle was measured using a Contact Angle Meter (XG-CAM). The specific heat capacity of paraffin was determined using Laser Flash Analysis (LFA467 type) from Netzsch Company (Selb, Germany).

### 2.2. Measurement Methods

**Figure 1:**
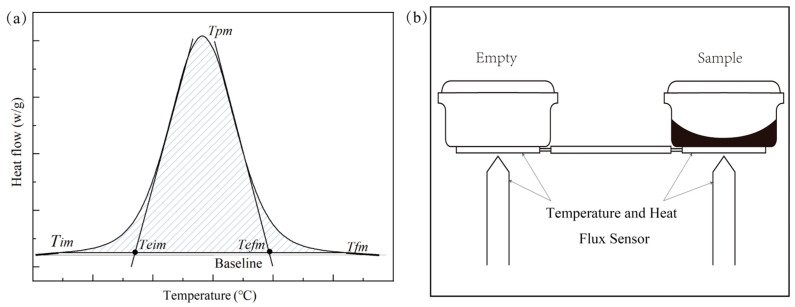
(**a**) The typical DSC curve; (**b**) a diagram of the DSC testing principle.

A heat-flow DSC was used to measure the thermal properties of paraffin with reference to the Chinese National Standard GB/T 19466.3-2004 [28]. An aluminum crucible was used as a sample container for measurement, nitrogen was used as a purge gas and a protective gas, and mechanical cooling was used for cooling. A temperature increase rate and an atmosphere flow rate were set. The temperature program of the instrument was as follows: cool down from 20 to −25 °C, hold at a constant temperature for 10 min, then raise the temperature from −25 to 25 °C, and hold at a constant temperature for 10 min. The first set of measured data was not adopted, as it was susceptible to the thermal history of the instrument. The sample mass was 5 mg to 20 mg, according to the instrument’s testing manual for conducting phase-transition process tests, including the *T_eim_* (the intersection point between the tangent of the maximum point of the front slope of the endothermic peak on the DSC curve and the baseline), *T_pm_* (the temperature of the maximum heat flow), *T_efm_* (the intersection point between the tangent of the maximum point of the end of the endothermic peak on the DSC curve and the baseline), as well as phase-transition enthalpy measurements. Additionally, in accordance with national standards, this paper adopts *T_eim_* as the melting temperature. The area integral on the DSC curve and the baseline is the latent heat of the PCMs, as shown in Figure 1a.

### 2.3. Instrument Correction

In the DSC measurement process, there are deviations between the detected sample temperature and the actual sample temperature due to factors such as the thermal conductivity of the sample pan (e.g., tantalum), thermal hysteresis between the sample pan and the thermocouple, the performance of the thermocouple, and the thermal conductivity of the atmosphere. Therefore, the instrument must be calibrated before measurement. Performing the temperature correction and sensitivity correction on the DSC instrument helps to reduce the drift of the measurement curve. Thus, the temperature parameters and phase-change values obtained from the analysis of the curve are closer to the theoretical values, which reduces the measurement uncertainty of the instrument.

**Figure 2:**
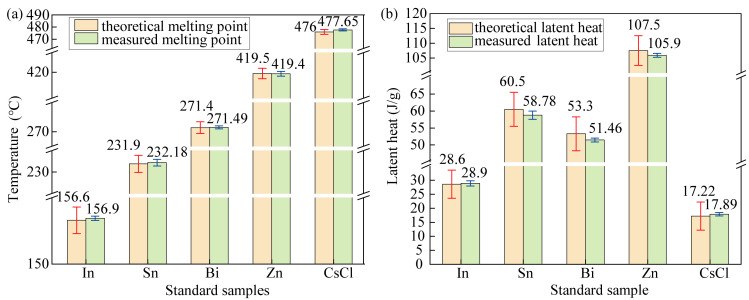
The theoretical and measured values of the thermal property parameters of the standard samples: (**a**) the theoretical and measured values of the melting point of the standard samples; (**b**) the theoretical and measured values of the latent heat of the standard samples.

The five standard samples, In, Sn, Bi, Zn, and CsCl, were measured in sequence. Each standard sample was measured three times. The data of the first group were susceptible to the thermal history of the instrument, so the average values and standard deviations of the data from the last two measurements were calculated to determine the measurement uncertainty range. According to the Class A instrument measurement performance requirements in the JJG936-2012 differential scanning calorimeter verification procedures, the allowable temperature indication uncertainty is ±2 °C, and the allowable thermal indication uncertainty is ±5 J/g [29]. The allowable measurement uncertainty ranges were established based on the theoretical melting points and latent heats of the standard samples, as shown in Figure 2. The figure demonstrates that the maximum deviation between the theoretical melting points and measured melting points of the five tested standard samples is +1.65 °C, while the maximum deviation in the phase-change latent heat is −1.84 J/g, with all measurement uncertainty falling within the allowable uncertainty. After performing temperature calibration and sensitivity calibration on the instrument, the measurement results and measurement uncertainty of the standard samples are in line with the requirements of the measurement performance of a Class A instrument, which means the instrument meets the requirements of the A grade measurement.

## 3. Experimental Results and Analysis

### 3.1. Sample Mass

**Figure 3:**
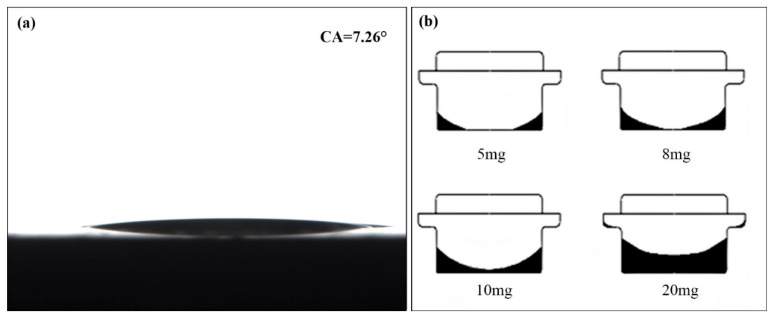
The contact angle between the paraffin wax and the aluminum crucible at 25 °C: (**a**) the contact angle between paraffin and aluminum; (**b**) the liquid surface depression of paraffin in the crucible with a diameter of 8 mm with different masses in the crucible.

Sample mass has a great influence on the experimental results. Paraffin wax is a non-polar liquid with excellent wettability, exhibiting a contact angle of 7° on the flat surface of the aluminum crucible material, as shown in Figure 3a. When the infiltration force between the liquid molecule and the solid (container wall) is greater than the cohesive force inside the liquid molecules, the liquid surface depression in the aluminum crucible is more serious. According to the reference values for the mass of the samples with 5–20 mg provided by the manufacturer, the distribution conditions of the liquid paraffin in the 8 mm diameter crucible, when the mass of the paraffin was 5 mg, 8 mg, 10 mg, and 20 mg, is shown in Figure 3b. As shown in Figure 3b, the state of the liquid surface depression of the sample worsen with the decrease in mass. When the sample mass increased to 20 mg, it can be clearly observed that some of the liquid in the crucible has “climbed” to the upper edge along the crucible wall surface, making the measurement more precise.

**Figure 4:**
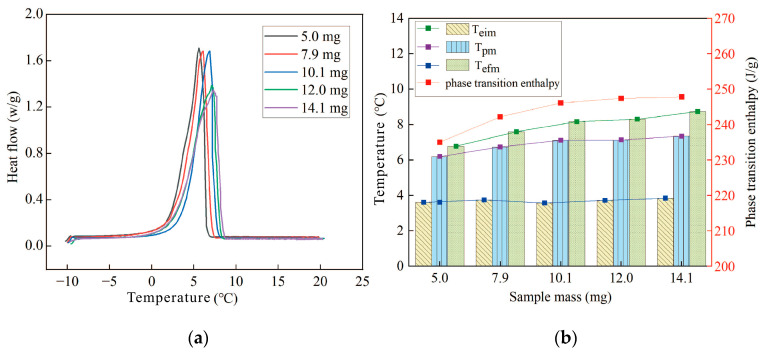
The thermal performance with different masses: (**a**) DSC curves for different masses; (**b**) the trend of changes in the thermal performance parameters of samples with different masses.

When testing the effect of sample mass on the experimental results, five groups with masses of 5.0, 7.9, 10.1, 12.0, and 14.1 mg were measured for thermal properties. Each group was measured twice. The first set of data, which was easily affected by the thermal history of the instrument, was excluded, and only the second set of test data was selected for analysis as the experimental results. In addition, the experimental heating rate was 1 °C/min, the protective gas was 100 mL/min, and the purge gas was 50 mL/min. The thermal performance parameters of different quality samples were also analyzed, as shown in Figure 4.

As seen from Figure 4a, the DSC curves gradually shift to higher temperatures as the sample mass increases. As the mass of the sample increases, the thickness of the ample increases, leading to an increased temperature gradient within the sample. Additionally, from Figure 1a, it can be observed that during dynamic testing by DSC, the sensor measures the sample temperature by contacting the bottom of the crucible through the holder. Due to the low thermal conductivity of the material, a temperature gradient exists, and a higher heat capacity increases this internal temperature gradient. This causes the temperature reading from the bottom sensor to be higher than the actual average temperature of the sample, resulting in a delay between the measured signal and the true temperature. Therefore, as the sample mass increases, thermal lag becomes more pronounced. A further analysis of the *T_eim_* in Figure 4b shows that the average value is 3.696 with a standard deviation of 0.0963, indicating low data dispersion, and the sample mass has no significant impact on *T_eim_*. Both *T_pm_* and *T_efm_* increase with the sample mass, with *T_efm_* showing a greater increase. The difference in *T_efm_* between the sample with a mass of 5.0 mg and the sample with a mass of 14.1 mg is 1.97 °C, and further increases in mass will exceed the temperature indication measurement uncertainty of Class A instruments.

As shown in Figure 4b, the phase-transition enthalpy of the samples increases with sample mass, and when the sample mass is less than 10.1 mg, the enthalpy increase is more significant. The phase-change enthalpy difference between the 5.0 mg and 14.1 mg samples is 12.8 J/g. The enthalpy difference for samples concentrated between 5.0 and 10.1 mg is 11.1 J/g, which accounts for 86.7% of the total difference. Both enthalpy differences exceed the thermal indication measurement uncertainty of Class A instruments. Figure 3b shows that when the sample mass is smaller, the material tends to distribute unevenly in the crucible, with more of the sample adhering to the wall, leading to greater heat loss due to heat exchange with the furnace body, and a large empty area at the bottom of the crucible, resulting in missing heat-flow signals and a lower measured phase-transition enthalpy. Therefore, when the sample mass is less than 10 mg, the uneven distribution in the crucible becomes the main source of measurement uncertainty, causing the results to exceed the indication uncertainty limits of Class A instruments.

Schawe et al. described the heat flow *Φ* entering the sample in a DSC experiment under a constant heating rate as the sum of two components: sensible heat ${\Phi \;}_{sen}$ and latent heat ${\Phi \;}_{let}$ [30]:

$$\Phi \;\left(T,\tau \right)={\Phi \;}_{sen}+{\Phi \;}_{let}$$

$$\Phi \;\left(T,\tau \right)=mc_{p}\beta +m∆h_{r}\frac{d\alpha }{dt}$$

The sensible heat flow is related to the sample mass *m*, specific heat capacity$\;c_{p}$, and the sample’s temperature change rate *β*, while the latent heat depends on *m*, the reaction enthalpy $∆h_{r}$, and the rate of internal changes as the system moves away from equilibrium $\;\frac{d\alpha }{dt}$. In practice, DSC measurements cannot separate these two heat-flow components. As the sample mass increases appropriately, the relative measurement uncertainty caused by sample uniformity decreases. However, as the sample mass increases further, thermal lag caused by heat transfer within the instrument and the sample leads to the heat flow continuing even after the actual melting temperature is exceeded. This results in an overestimation of the latent heat, as the measured latent heat includes the sensible heat from the thermal lag region.

**Figure 5:**
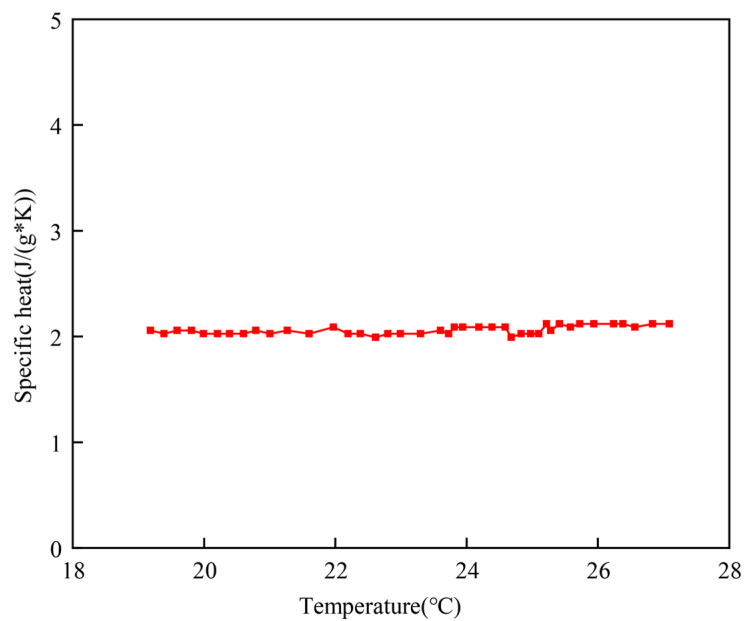
Specific heat capacity of paraffin measured by LFA.

As shown in Figure 5, the specific heat capacity of liquid RT5 is approximately 2.07 J/g*K. Compared to the sample with a mass of 10.1 mg, the *T_efm_* of the sample with a mass of 14.1 mg is delayed by 0.57 °C, with the corresponding sensible heat calculated as 1.179 J/g. The difference in measured latent heat between these two samples is only 1.7 J/g. Therefore, when the sample mass exceeds 10 mg, the sensible heat resulting from thermal lag becomes the primary source of measurement uncertainty in the latent heat measurement.

### 3.2. Heating Rate

**Figure 6:**
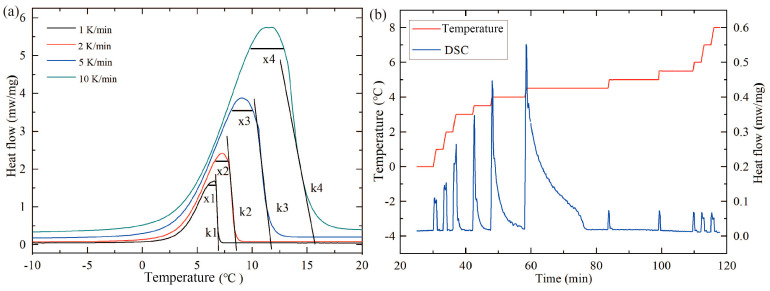
DSC curves with different measurement modes: (**a**) DSC curves with different heating rates by the dynamic mode; (**b**) DSC curve measured by the step mode.

When investigating the effect of heating rate on the experimental results, the sample mass was controlled at 10.2 mg, with a protective gas flow rate of 100 mL/min and a purge gas flow rate of 50 mL/min. Two heating modes, dynamic mode and distributed mode, were used for testing the sample. In the dynamic mode, the thermal properties of the sample were measured at heating rates of 1, 2, 5, and 10 °C/min, while the measurement was performed at a heating rate of 1 °C/min in the distributed mode. Each group was measured twice. The second set of test data was selected for analysis as the experimental results, and the resulting DSC curve is shown in Figure 6.

As seen in Figure 6a, the four DSC curves obtained at different heating rates have basically the same *T_eim_*, but the *T_pm_* and *T_efm_* are greatly influenced by the heating rate. As the heating rate increases, the *T_pm_* shifts significantly towards higher temperatures, and the slopes k1, k2, k3, and k4 after the peak gradually increase. Therefore, the range of the phase transition determined by the *T_eim_* and *T_efm_* becomes larger. With the increase in heating rate, the temperature gradient inside the sample increases, resulting in a more pronounced thermal lag effect. Secondly, owing to the substantial thermal resistance and heat capacity of the sample, it exhibits significant thermal inertia, resulting in a delayed response to temperature changes. Furthermore, as the heating rate increases, materials with high thermal inertia demonstrate a comparatively lower temperature response. Holba [31] summarized the descriptions and derivation processes of the thermal inertia term in the related literature, indicating that the thermal inertia term extends to all types of DSC and DTA measurements.

Additionally, when the heating rate is 1 °C/min, the curve at the peak is sharp, with peak separation observed. As the heating rate increases, the widths of the phase-change peak x1, x2, x3, and x4 gradually increase, and the peak separation disappears. This is because paraffin is a mixture of straight-chain alkanes of similar melting points; during dynamic mode testing, when the heating rate is lower, the components of the mixture have more time to undergo phase transitions as they reach their respective phase-transition temperatures. This causes the heat-flow signal measured by the DSC become more dispersed as the temperature increases, narrowing the width of the phase-change peak, leading to the phenomenon of peak separation. As the heating rate increases, the temperature gradient inside the sample becomes larger, causing more components to be in thermal and phase-transition disequilibrium. This thermal exchange and phase transition continue beyond the peak point, leading to an increase in peak width x with increasing heating rates.

As shown in Figure 6b, compared to the dynamic measurement mode, the DSC curve of paraffin obtained in the step mode consists of a series of peaks with varying heights. This is because each component of the paraffin undergoes a phase transition at its corresponding phase-transition temperature, and with sufficient time, the phase transition is completed at that temperature, after which the heat-flow signal returns to the baseline. In this mode, the PCM eventually reaches a thermally saturated state during each isothermal step, which more accurately reflects the phase-transition temperature range of the material under ideal conditions. Although the step mode measurement allows for better thermal equilibrium within the material, the characterization conditions of the PCM’s physical properties are inherently tied to its practical application. Therefore, while the phase-transition temperature range obtained through step mode can serve as a valuable reference, the corresponding phase-transition temperature range should be characterized based on the heating rate relevant to the actual application of the paraffin in the dynamic mode.

### 3.3. Atmosphere Flow

**Figure 7:**
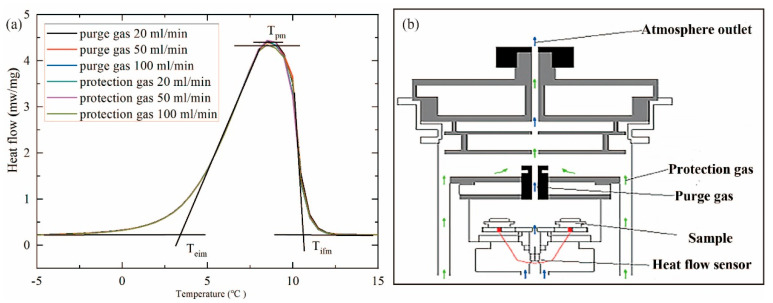
DSC measurements with different atmosphere flow rates: (**a**) atmosphere flow chart in DSC; (**b**) the DSC curves with different atmosphere flow rates.

Atmospheric flow is one of the key factors affecting the experimental results. The sample mass was controlled at 10.3 mg, the heating rate was set to 2 °C/min, and the flow rates of the purge gas and the shielding gas are controlled, respectively. The flow rate of the purge gas and the protective gas were set to 20, 50, and 100 mL/min, respectively. Then, the effect of atmospheric flow on the DSC curves of paraffin was investigated. Similarly, each group was tested twice, and the results from the second test were selected for analysis. The test results are shown in Figure 7a, and the obtained phase-change latent heat of the samples is shown in Table 1.

As seen from Figure 7a, the six curves of DSC obtained by changing the flow rate of the purge gas and the shielding gas, respectively, stay highly consistent. In the front and back of the wave peak, the six DSC curves are basically the same. In addition, the *T_eim_*, *T_pm_*, and *T_efm_* of the six DSC curves obtained from the analysis curve are also nearly identical. It indicated that changing the flow rate of the atmosphere has no effect on the *T_eim_*, *T_pm_*, and *T_efm_* of the phase transition. The analysis of the latent heat of the phase change for the six DSC curves shows that when the purge gas flow increase, the latent heat decreases with the uncertainty remaining within ±5 J/g. However, with the increase in shielding gas flow, the latent heat of the phase change does not change significantly.

As seen from Figure 7b, purge gas is introduced into the furnace of the DSC instrument, while the protective gas typically passes through the periphery of the furnace body and flows out of the small holes on the outermost cover of the furnace body. The role of protective gas is to protect the heating furnace body and extend its working life. Since there is no direct contact with the sample in the furnace, the protective gas has no effect on the measurement results. On the other hand, the purge gas enters through the bottom of the sample holder inside the furnace body and flows out of the small hole on the innermost cover of the furnace body to directly contact the sample in the innermost furnace body, thereby affecting the measurement results.

The inert gas nitrogen is used as the purge gas. Due to the influence of the heat conduction of the gas, the contact thermal resistance is increased, the thermocouple’s sensitivity to temperature decreases, and the sensitivity coefficient of the instrument is reduced. In the analysis of the DSC curve, the sensitivity coefficient is obtained by dividing the measured signal integrated area of the melting section by the melting heat enthalpy. When the sensitivity coefficient of the instrument is reduced, the sensitivity coefficient of the given temperature of the instrument is higher than the actual value, leading to a lower analysis result for the latent heat of the phase change.

## 4. Comparison of Recent Research

Liu et al. [32] conducted experiments on the effects of heating rate and sample mass on paraffin-based composite phase-change materials using DSC. The shift in phase-change peaks indicates a correlation between the hysteresis of the DSC curves and the rate of temperature change, where higher heating rates result in a broader temperature range, consistent with the findings of this study. Notably, larger samples may lead to deviations in the DSC curves due to their higher interfacial free energy. However, a quantitative analysis of the impact of excessively large or small sample masses on test results has yet to be conducted. In addition to this analysis, the study highlights that the good wettability between paraffin and aluminum crucibles can lead to uneven distribution when the mass is too small, thereby providing more reliable test quality parameters during the early stages of material testing. John et al. [20] conducted a measurement analysis through orthogonal experiments, determining that a heating rate below 0.5 °C/min, an isothermal hold of 5 min, a sample mass of less than 10 mg, and a working temperature range of ±20 °C allow for the accurate measurement of the enthalpy and initial temperature of dodecane and n-hexadecane. This finding is consistent with the variable analysis results presented in this paper. However, this study extends the investigation by conducting experiments based on these factors and performing an in-depth analysis of the underlying mechanisms, thereby enhancing the general applicability of the experimental results and facilitating their extension to other materials. Sun et al. [33] investigated the testing conditions for n-octadecane paraffin and hexahydrated calcium chloride. Their results indicated that, with increasing heating rates and sample masses, the range of phase-transition temperatures expanded, although no effects on phase-change latent heat were observed. This study primarily analyzes the impact of various measurement parameters—such as instrument calibration prior to testing, sample distribution in the crucible, sample mass, heating rate, atmospheric flow rate, and the structural characteristics of the testing apparatus—on the measurement results. We provide a comprehensive exploration of how to select appropriate measurement parameters based on the material properties when using DSC instruments to determine phase-transition parameters, thereby enhancing the accuracy of the results and offering valuable insights for future research.

## 5. Conclusions

In this study, a large number of thermal performance measurement experiments were carried out on paraffin wax RT5 using heat-flux DSC. It was found that parameters such as sample mass, instrument heating rate, and atmosphere flow rate have a certain influence on the thermal performance results of the sample. The experimental results show that when the sample mass is less than 10 mg, the uneven distribution of paraffin in the crucible is the main source of measurement uncertainty in the measurement of phase-transition enthalpy. When the sample mass is greater than 10 mg, the main measurement uncertainty is due to the inclusion of sensible heat associated with thermal lag. The former measurement uncertainty is relatively larger. Therefore, it is recommended to use a sample mass of 10 mg for the thermal performance measurements of paraffin materials when the crucible diameter is 8 mm. When the heating rate is used as the experimental variable, as the heating rate increases, the DSC curve shifts backward due to the effects of thermal lag and thermal inertia. It is recommended to select a heating rate consistent with the material’s application scenario for dynamic mode measurements. This approach can more accurately provide valuable insights for further research into the material’s applications. When atmosphere flow rate is used as the variable, changing the protective gas flow rate during measurement has no effect on the results. However, increasing the flow rate of the purge gas leads to a relative decrease in the measured phase-transition enthalpy. Therefore, a lower atmosphere flow rate should be maintained throughout the entire testing process.

## Figures and Tables

**Table 1:** Thermal properties of DSC curves measured by different atmosphere flows.

Atmosphere Name	Atmosphere Flow (mL/min)	*_Teim_* °C	*T_pm_* °C	*T_efm_* °C	Melting Enthalpy J/g
protection gas N_2_	20	3.81	8.61	10.67	243.0
protection gas N_2_	50	3.85	8.68	10.70	242.7
protection gas N_2_	100	3.81	8.54	10.61	243.2
purge gas N_2_	20	3.78	8.61	10.70	246.1
purge gas N_2_	50	3.83	8.58	10.60	245.2
purge gas N_2_	100	3.81	8.54	10.61	243.2

## Data Availability

The original contributions presented in the study are included in the article, further inquiries can be directed to the corresponding author.

## Abbreviations

DSC	Differential scanning calorimeter
PCM	phase-change material
*T_eim_*	extrapolated onset temperature
*T_efm_*	extrapolated final temperature
*T_pm_*	melting peak temperature
PCES	phase-change energy storage
EPCM	encapsulated phase-change material
CA	contact angle
*Φ*	heat flow
$\Phi_{sen}$	sensible heat
$\Phi_{let}$	latent heat
*m*	mass
$c_{p}$	specific heat capacity
*β*	temperature change rate
$∆h_{r}$	reaction enthalpy
$\frac{d\alpha }{dt}$	the rate of internal changes as the system moves away from equilibrium
*k*	slope
x	width
